# Understanding the Association between Environmental Factors and Longevity in Hechi, China: A Drinking Water and Soil Quality Perspective

**DOI:** 10.3390/ijerph15102272

**Published:** 2018-10-16

**Authors:** Qucheng Deng, Lijuan Chen, Yongping Wei, Yonghua Li, Xuerong Han, Wei Liang, Yinjun Zhao, Xiaofei Wang, Juan Yin

**Affiliations:** 1 School of Earth and Environmental Sciences, the University of Queensland, Brisbane 4072, Australia; uqqdeng@uq.edu.au (Q.D.); ljchen@lzb.ac.cn (L.C.); yongping.wei@uq.edu.au (Y.W.); 2 Key Laboratory of Ecohydrology of Inland River Basin, Northwest Institute of Eco-Environment and Resources, Chinese Academy of Sciences, Lanzhou 730000, China; 3 Key laboratory of Environmental Change and Resources Use in Beibu Gulf, Guangxi Teachers Education University, The Ministry of Education, Nanning 530001, China; crpp0104@163.com; 4 Institute of Geographical Sciences and Natural Resources Research, Chinese Academy of Sciences, Beijing 100101, China; 5 Guangxi Zhuang Autonomous Region Environmental Monitoring Center, Nanning 530028, China; amelly@yeah.net (X.H.); wangxiaofei26@163.com (X.W.); 6 Guangxi Environmental Information Center, Nanning 530028, China; liangwei_gxepb@163.com (W.L.); 7 School of Geography and Planning, Guangxi Teachers Education University, Nanning 530001, China; 8 Department of Management Science and Engineering, Guangxi University of Finance and Economics, Nanning 530003, China; yinjuan101@163.com

**Keywords:** Hechi, regional longevity, drinking water, soil, trace element

## Abstract

The aging population is a big challenge all over the world. However, there are few studies to date investigating the effects of trace element and mineral levels in drinking water and soil (especially in karst areas) on longevity. This study aims to examine temporal and spatial variations in longevity in Hechi (which is recognized as a longevity city) and to investigate relationships between longevity and trace element and mineral levels in drinking water and soils in this city (the karst landscape). Population data were collected from relevant literature and four national population censuses in 1982, 1990, 2000 and 2010. Drinking water and soil samples from Hechi were collected and analyzed. The results demonstrated an obvious clustered distribution for the longevity population in Hechi that has existed stably for decades. The longevity index tended to be significantly positively correlated with H_2_SiO_3_, Ca and Fe in drinking water and significantly negatively correlated with Sr in soil, indicating that drinking water characteristics contributed significantly to the observed regional longevity. The karst landscape is responsible for abundant trace elements in underground rivers in Hechi, which are beneficial to human health when consumed as drinking water. Good quality and slightly alkaline drinking water rich in trace elements such as H_2_SiO_3_, Ca, Fe, Na, Mg and low in heavy metals such as Pb and Cd might be an important factor contributing to the longevity phenomenon in Hechi.

## 1. Introduction

Aging is a big challenge all over the world because of the substantial socio-economic impacts associated with the aging population. The United Nations defines a country with more than 10% of its population over 60 years old as an aging society [1]. China became an aging society in 2000 and is now one of the world’s fastest aging countries. According to the National Population Census in China in 2010, the number of people over 60 in this country has reached approximately 178 million, representing nearly 13.26% of the total population [2,3]. Therefore, as a country with a large elderly population, China will undergo significant changes in its age structure, and their impacts may exceed those of other countries in the future. 

The geographic distribution of the elderly population in China is uneven, with centenarians mainly clustered in southern regions. In particular, Guangxi in southern China was recognized in 2010 as the province with the leading centenarian prevalence in Mainland China. Moreover, the city of Hechi in Guangxi was identified in 2016 as a longevity city, and Bama, a county in Hechi, has been considered since 1991 to have one of the most long-lived populations globally. Thus, Hechi can be selected as a typical longevity city for investigating important factors underlying longevity, and such research is useful for developing a healthy aging society worldwide.

It is widely known that longevity is affected by different factors. Most studies thus far have mainly focused on the aspect such as environmental factors [4,5], socioeconomic factors [6,7], medical factors [8], and genetic factors [8,9,10]. Among them, environmental factors that potentially impact human health have been studied intensively, including climate [4,11], air quality [12,13], natural and drinking water quality [14,15,16,17], and soil quality [18,19,20]. 

Specifically, the trace elements and mineral nutrients present in drinking water and soil have attracted much attention. This is because trace elements and minerals in drinking water provide more assimilated elements for humans compared with those obtained through food [16,21], and essential trace elements and minerals in soil affect drinking water quality and food, with indirect impacts on human health [18,22]. Thus, many researchers have examined the impacts of these components in drinking water and soil on human health as well as their indirect influence on human health and regional longevity. For instance, Olawoyin et al. assessed the levels of heavy metals in the soil environment along the Niger River area and their potential risks to human health. They determined that Pb and Cr might be harmful to humans when their concentrations reach certain levels in the soil environment [19]. Liu et al. studied the associations between the chemical composition of water and longevity in different regions within Xinjiang Province and reported that high levels of total nitrogen and the macro elements Mg and Ca may be beneficial for health [16]. Additionally, Chowdhury et al. examined the levels of heavy metals in drinking water in many developing countries and found that exposure to heavy metals, such as As, Pb and Cd, could threaten human health [15]. 

Despite the useful information provided by these studies, they merely focused on one specific aspect or certain elements in drinking water or soil. Because the impacts of trace elements and mineral nutrients in drinking water and soil on human health act in a combined, integrated or interactive way, previous studies have failed to provide a systematic understanding of the mechanism by considering how trace elements, mineral nutrients from both drinking water and soil environment impact on the regional longevity comprehensively. In addition, landscapes, which can strongly influence regional water and soil quality, differ dramatically, and trace elements and mineral nutrients in Hechi (karst region) would be especially unique compared with other longevity areas. Thus, knowledge of the effects of trace element and mineral nutrient levels in drinking water and soil on the longevity under this unique landscape, as well as their inter-relationships, is extremely important.

This study aims to understand relationships between the longevity phenomenon and trace element and mineral levels in drinking water and soil under Hechi’s karst landscape. To this end, two longevity indicators, namely, the centenarian prevalence and longevity index, were chosen to investigate temporal and spatial variations in longevity in eleven counties of Hechi for the 1982–2010 period. Drinking water and soil were sampled for analysis of Hechi as a whole and for differences among longevity and non-longevity counties. Finally, correlation between trace element and mineral levels and the two longevity indicators and how the karst landscape contributes to differences in trace element and mineral levels among the counties were also analyzed. We hope the results will improve our understanding of how natural environmental factors influence regional longevity.

## 2. Materials and Methods 

### 2.1. Case Study Area Description

**Figure 1:**
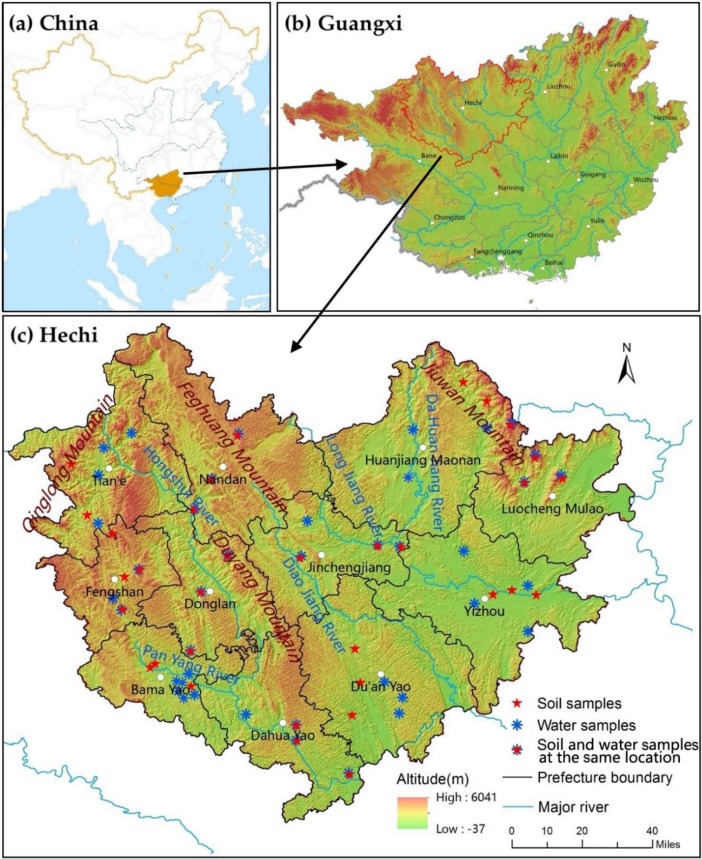
Location of Hechi and its sampling sites(**c**) in Guangxi (**b**), China (**a**).

Hechi is situated in northwest Guangxi, China (Figure 1), with an area of approximately 33,500 km^2^. Karst mountain regions account for approximately 65% of the total area of Hechi [23]. All counties in Hechi have rocky areas, but the counties with the most rocky areas are Duan, Fengshan, Bama, Donglan and Dahua. The topography is higher in the northwest region and lower in the southeast region. Jiuwan Mountain is located in the northeast, Fenghuang Mountain is located in the northwest and Duyang Mountain is located in the middle. The Panyang River in the Red River basin, which is the primary river in Hechi, passes through longevity counties, including Bama, Donglan and Fengshan, and serves as the primary drinking water resource for these three counties. Hechi is located within the subtropical climate zone, and the annual temperature and rainfall are 19.24–20.79 °C and 1194–1601 mm, respectively. 

Hechi has a population of approximately four million, and its centenarian prevalence reached 17.9 in 2016, which is one of the highest cities in China [24]. Hechi comprises 11 counties, including Jinchengjiang, Huanjiang, Nandan, Tian’e, Donglan, Fengshan, Bama, Duan, Dahua, Luocheng and Yizhou. In recent years, six of these 11 counties, Bama, Tian’e, Fengshan, Donglan, Dahua and Yizhou, have been classified as ‘Chinese longevity areas’ due to their significant proportions of centenarians [24]. Hechi is located in a relatively isolated area due to its slow economic development. Health and longevity in this city might be associated with the local geographical environment and consequently and more strongly affected by natural environmental factors, such as air, soil, drinking water, vegetation and food characteristics, as opposed to external factors [24]. This situation is unique and has the ability to reveal relationships between longevity and the environmental factors. 

### 2.2. Population and Environmental Data Collection

Chinese demographical data for 1982, 1990, 2000 and 2010, the most representative data recognized by many researchers [25], were collected [26,27,28,29] to analyze the longevity phenomenon in Hechi. The centenarian prevalence, which is the proportion of centenarians per 100,000 inhabitants, is used because it can reflect extreme longevity in a total population in a given area and enable comparisons with other areas. The longevity index, which is calculated as the ratio of the population greater than 90 years to the population over 65 years, is also employed to reflect the relative elderly population ratio [30]. The longevity index represents the continuity of longevity, and the centenarian prevalence, together with the longevity index, can be used to assess the longevity phenomenon in an area and the structure of the population.

In September 2015, 40 drinking water samples and 33 soil samples were collected from the 11 counties of Hechi (Figure 1). Trace elements and mineral nutrients potentially impacting human health were chosen for analysis based on the China National Drinking Water Quality Standard (GB5479-2006) [31], the World Health Organization recommended standard (WHO-Guideline 2005) [32], and the China National Soil Quality Standard (GB15618-2008) [33]. Parameters measured were pH, arsenic (As), calcium (Ca), cadmium (Cd), chromium (Cr), copper (Cu), iron (Fe), potassium (K), lithium (Li), magnesium (Mg), manganese (Mn), molybdenum (Mo), sodium (Na), nickel (Ni), phosphorus (P), lead (Pb), selenium (Se), strontium (Sr), zinc (Zn), sulfate ion (SO_4_^2−^) and silicic acid (H_2_SiO_3_) in the drinking water and aluminum (Al), Ca, Fe, K, Mg, Na, As, Cd, cobalt (Co), Cr, Cu, Li, Mn, Mo, Ni, P, Pb, sulfur (S), Se, Sr and Zn in soil. 

### 2.3. Statistical Analysis

Population data were collected from the third, fourth, fifth and sixth National Population Census in 1982, 1990, 2000 and 2010. Longevity indicators, i.e., the centenarian prevalence and longevity index, were calculated using SPSS 22.0 (IBM, New York, NY, USA). ArcGIS 10.5.1 (ESRI, Redlands, CA, USA) was utilized to determine the spatial autocorrelation of the two longevity indicators for 1990–2010. ArcGIS 10.51 was then applied to produce the graphs of the spatial distributions of the longevity indicators. Trend Surface Interpolation of ArcGIS was used to reflect the trace element levels in drinking water and soil in Hechi, as this method could more precisely reflect the relationship among sampling points. We have used the Kolmogorov-Smirnov to determine whether the data were normally distributed. For the data were not normally distributed, we used logarithmic transformation to normalize the distributions. In addition, Pearson correlation analysis was conducted to detect relationships between the level of trace elements and mineral level and longevity indicators.

The Moran index, Moran’s *I*, was applied to detect the spatial autocorrelation of the two longevity indicators and was calculated using the following formula [34]:

$$I=\frac{n\sum_{i=1}^{n}\sum_{j=1}^{n}w_{ij}\left(x_{i}-\bar{x}\right)\left(x_{j}-\bar{x}\right)}{(\sum_{i=1}^{n}\sum_{j=1}^{n}w_{ij})\sum_{i=1}^{n}{\left(x_{i}-\bar{x}\right)}^{2}}$$

where *i* and *j* indicate the spatial unit number *n*, and *x* is the variable of interest. *x_i_* and *x_j_* in the formula refer to the values of the variable at positions *i* and *j*. In addition, $\bar{x}$ refers to the mean of *x*; the weights *W_ij_* in the formula are presented as a (*n* × *n*) weight matrix. The weight matrix describes the association between an individual element and the other elements around it, and weight is based on the contiguity relationship. Moran’s *I* values typically range from −1 to +1. A positive Moran’s *I* value denotes positive spatial autocorrelation, whereas a negative value denotes negative spatial autocorrelation. For statistical hypothesis testing, values of Moran’s *I* were evaluated based on *Z*-score. A |*Z*| value higher than 1.96 indicates significant spatial autocorrelation at the 0.05 significance level, and a |*Z*| value higher than 2.58 indicates significant spatial autocorrelation at the 0.01 significance level. 

## 3. Results

### 3.1. Temporal and Spatial Distributions of the Longevity Population in Hechi

#### 3.1.1. Regional Differences during 1982–2010

**Figure 2:**
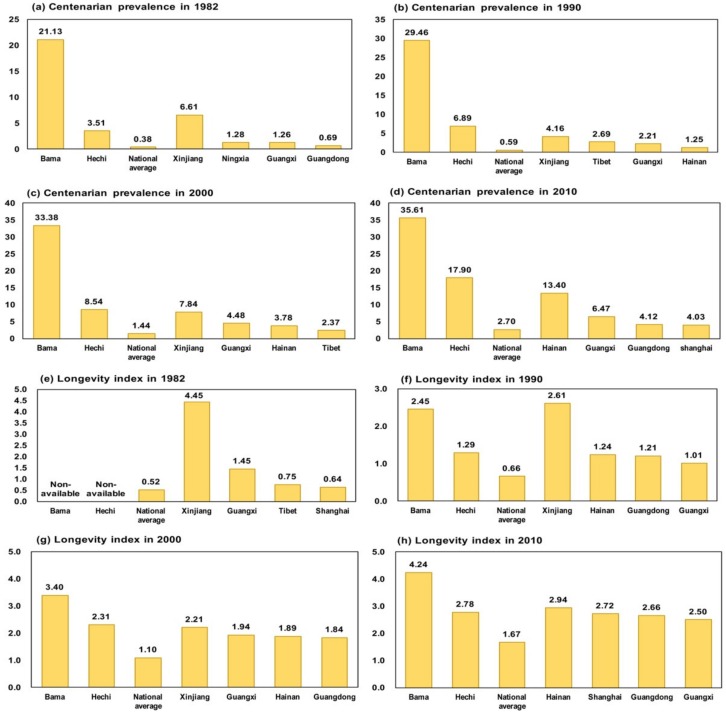
Comparison of the centenarian prevalence in Hechi, Bama and the other top four provinces in China in 1982 (**a**), 1990 (**b**), 2000 (**c**) and 2010 (**d**) and comparison of the longevity index in Guangxi, Hechi, Bama and the other top four provinces in China in 1982 (**e**), 1990 (**f**), 2000 (**g**) and 2010 (**h**). (Data for the national average and top four provinces were obtained from Wang et al., 2015 [7]).

Figure 2a–h present the centenarian prevalence and longevity index in China, Hechi, Bama county and four other top provinces in China, as based on the population censuses of 1982, 1990, 2000 and 2010. The values for these two indicators increased significantly over time. For the centenarian prevalence, Figure 2a–d show that the centenarian prevalence in Hechi was 9.2 times higher than the national level in 1982, 11.7 times higher than that in 1990, 5.9 times higher than that in 2000 and 6.6 times higher than that in 2010. Hechi’s centenarian prevalence was higher than in all of the top four provinces from 1990 to 2010. Bama County maintained the highest centenarian prevalence across the population censuses. For example, Bama’s centenarian prevalence was more than 13.2 times higher than the national centenarian prevalence from 1982–2010. Moreover, the centenarian prevalence in Bama was significantly higher than the centenarian prevalence in Hechi and the other top four provinces. With regard to the results for the longevity index shown in Figure 2e–h, Hechi was close to the top four provinces during 1990–2010. Moreover, Bama County’s longevity index remained higher than both the national level and that of the other top four provinces. As a result, centenarian prevalence and longevity index in both Hechi and Bama remained relatively high over the past four decades.

#### 3.1.2. Temporal and Spatial Variations in the Centenarian Prevalence and Longevity Index during 1982–2010 in Hechi

**Figure 3:**
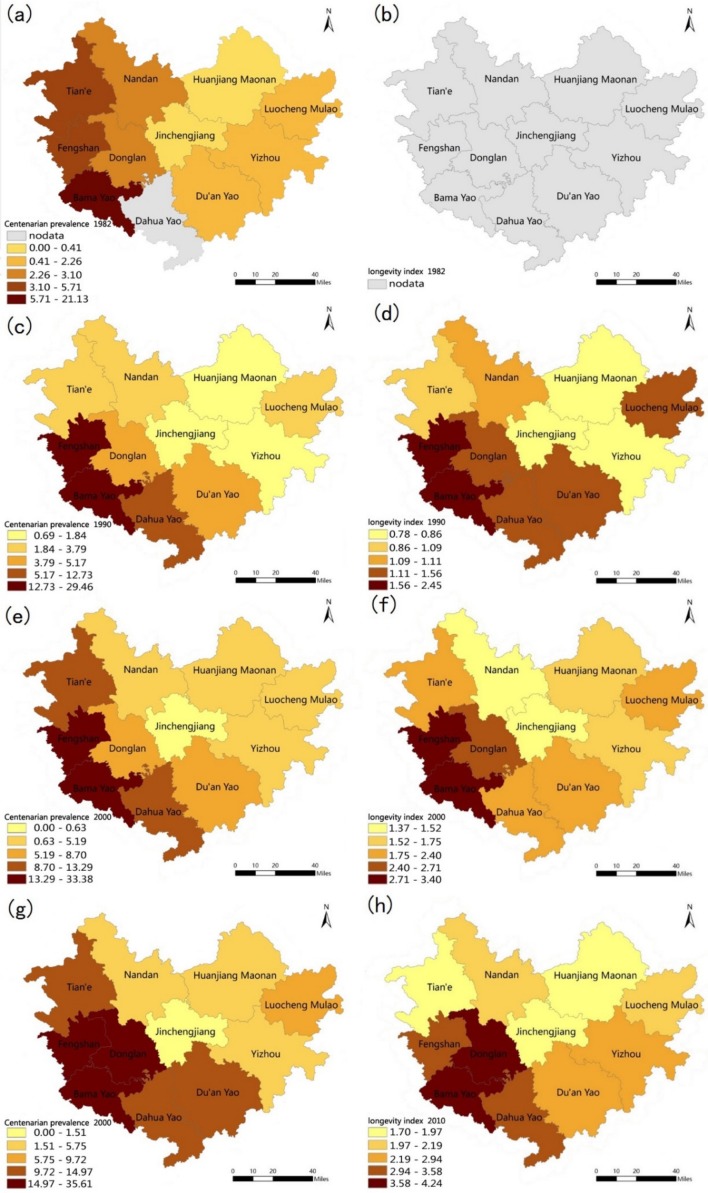
Distribution of the centenarian prevalence in 1982 (**a**), 1990 (**c**), 2000 (**e**) and 2010 (**g**) and the longevity index in 1982 (**b**), 1990 (**d**), 2000 (**f**) and 2010 (**h**) in Hechi.

Figure 3 depicts the temporal and spatial variations in the centenarian prevalence and longevity index at the county level in Hechi over the course of four decades. As indicated in Figure 3a,c,e,g, the distribution of the centenarian prevalence was heterogeneous among the counties. The counties with the highest centenarian prevalence were Bama, Fengshan and Donglan, which are all located within the core areas of the Panyang River Basin in the southwestern region of Hechi. Among the counties, the highest centenarian prevalence was found for Bama. Conversely, those areas with a lower centenarian prevalence were found to be in the northern and middle areas of Hechi, including Nandan, Huanjiang and Jinchengjiang. The data in Figure 3b,d,f,h show that the longevity index in Hechi has a similar distribution as that of the centenarian prevalence, with Fengshan, Bama and Donglan counties having a relatively high longevity index across the decades. The areas in the middle and northern areas of Hechi such as Jinchengjiang and Huanjiang maintained a relatively lower longevity index. Both the two longevity indicators in Hechi indicate that the southwestern regions had been a stable longevity area for the past decades. 

#### 3.1.3. Spatial Autocorrelation of Longevity Indicators during 1990–2010 in Hechi

The values of Moran’s *I* for the counties in Hechi from 1990 to 2010 are presented in Table 1. Moran’s *I* for the centenarian prevalence and longevity index were higher than 0.3, indicating relatively high spatial autocorrelations among the counties in Hechi with regard to the longevity phenomenon. Many of the *Z* values in this study for Hechi from 1990 to 2010 were higher than 1.96 and significant at the 0.05 confidence level, indicating spatial autocorrelation. The Moran’s *I* and Z values suggest that regional longevity in Hechi might be associated with the geography of the local natural environment.

### 3.2. Drinking Water and Soil Quality in Hechi

#### 3.2.1. Assessment of Drinking Water and the Soil Environment 

The quality of drinking water in Hechi is shown in Table 2. The results of the average value of trace element and mineral levels showed that all indicators satisfied the standards of drinking water quality in China and the guidelines of the WHO. The standard-reaching rates for Ca, Cd, Cr, Cu, Fe, K, Li, Mg, Mn, Mo, Na, Ni, P, Pb, Se, Sr, Zn, SO_4_^2−^ and H_2_SiO_3_ were all 100% for the sampling sites, whereas the pH and As level were 94.9% and 97.4%, respectively, of the standards. These results indicate that the drinking water quality in Hechi was high. In addition, as indicated by significant standard deviations, there were substantial regional differences in the levels of the trace elements Fe, Ca, Zn, SO_4_^2−^, and H_2_SiO_3_ in Hechi. For example, the longevity areas exhibited higher levels of trace elements such as Fe, Ca and H_2_SiO_3_, whereas non-longevity areas were lower in these trace elements but higher in trace elements such as Zn and SO_4_^2−^. 

The quality of soil in Hechi is presented in Table 3. The average values of cultivated soil in Hechi were all in accordance with the soil environmental quality standards of China (GB15618-2008). Among all sampling sites, nearly 70%, 82%, 88%, 91%, 94% and 97% of Cd, Zn, Cu, Cr, Ni and Pb levels, respectively, reached the standards. Other parameters such as As, Co, Se were 100% of the standard. Soil sample analysis indicated that soil quality in Hechi was generally good. In addition, the standard deviation among the soil sites sampled was relatively high. The contents of Cd, Zn, Cu, Cr, Ni and Pb in cultivated land were high in some areas of Hechi and closely related to the geological structure of Hechi. For example, the northeastern part of Hechi is in the non-ferrous metal mineralization zone, with high natural background levels of these metals.

#### 3.2.2. Differences in Trace Element and Mineral Levels in Drinking Water and Soil in Longevity and Non-Longevity Counties 

Despite the good quality of water in Hechi, there were still differences among trace element and mineral levels in longevity and non-longevity counties (Table 4). Levels of trace elements and minerals, such as H_2_SiO_3_, Ca, Na, Fe, Mg, and Li, in the longevity counties were relatively higher than were those in the non-longevity counties, e.g., H_2_SiO_3_ (1.8 times), Ca (1.1 times), Na (2.9 times), Fe (1.1 times), Li (2.2 times) and Mg (1.5 times). In contrast, Zn, Pb, Cd, Cu, Mn and SO_4_^2−^ levels in the longevity counties were lower than those in non-longevity counties by 0.2 times, 0.2 times, 0.3 times, 0.4 times, 0.03 times and 0.4 times, respectively. In addition, compared with the longevity counties (the average of Bama, Fengshan and Donglan), Bama showed higher trace element and mineral levels, such as H_2_SiO_3_, Na, Ca, Fe, Mg and Li, were 1.5 times, 1.5 times, 1.3 times, 1.2 times, 1.7 times and 2.0 times higher. 

As shown in Table 5, the soil environment in Hechi was generally good. There were fewer differences in soil environmental indicators than drinking water indicators between longevity and non-longevity counties. The major difference was that Sr levels in longevity areas are relatively lower (0.5 times) than those in non-longevity counties. Trace element levels of Zn, Mo, Cu, Co, and Fe in longevity counties were relatively higher than those in non-longevity counties, and compared with longevity counties (the average of Bama, Fengshan and Donglan), Bama had relatively low levels of Cd, Pb, Se, and Zn.

### 3.3. Correlations of the Centenarian Prevalence, Longevity Index and Mineral and Trace Element Levels in Water and Soil in Hechi

The results of the Pearson correlation analysis are shown in Table 6 and Table 7. Table 6 illustrates the relationship between trace element and mineral levels drinking water and longevity indicators. The H_2_SiO_3_ level in drinking water showed a significant positive correlation with both the centenarian prevalence and the longevity index (*r* > 0.42); Ca and Fe were found to be significantly correlated with the centenarian prevalence (*r* > 0.39), and Na, Mg, Li, Mo, Se were positively correlated with the centenarian prevalence and longevity index. However, Zn was significantly negatively correlated with the longevity index (**|***r***|** = 0.32), and Pb, Cd, SO_4_^2−^ and Mn were negatively correlated with the centenarian prevalence and longevity index (**|***r***|** > 0.20). 

Table 7 illustrates the relationship between trace element and mineral levels in soil and longevity. A significant negative correlation between Sr and the longevity index (**|***r***|** = 0.345) and a negative correlation between K and the centenarian prevalence and longevity index (**|***r***|** > 0.20) were found. Conversely, Mo, Na, Fe, Co, Zn, and Cu were positively correlated with the longevity index (*r* > 0.20). Based on Pearson correlation analysis, trace element and mineral levels in drinking water had a more direct and obvious impact on regional longevity than did in the soil environment in Hechi.

**Figure 4:**
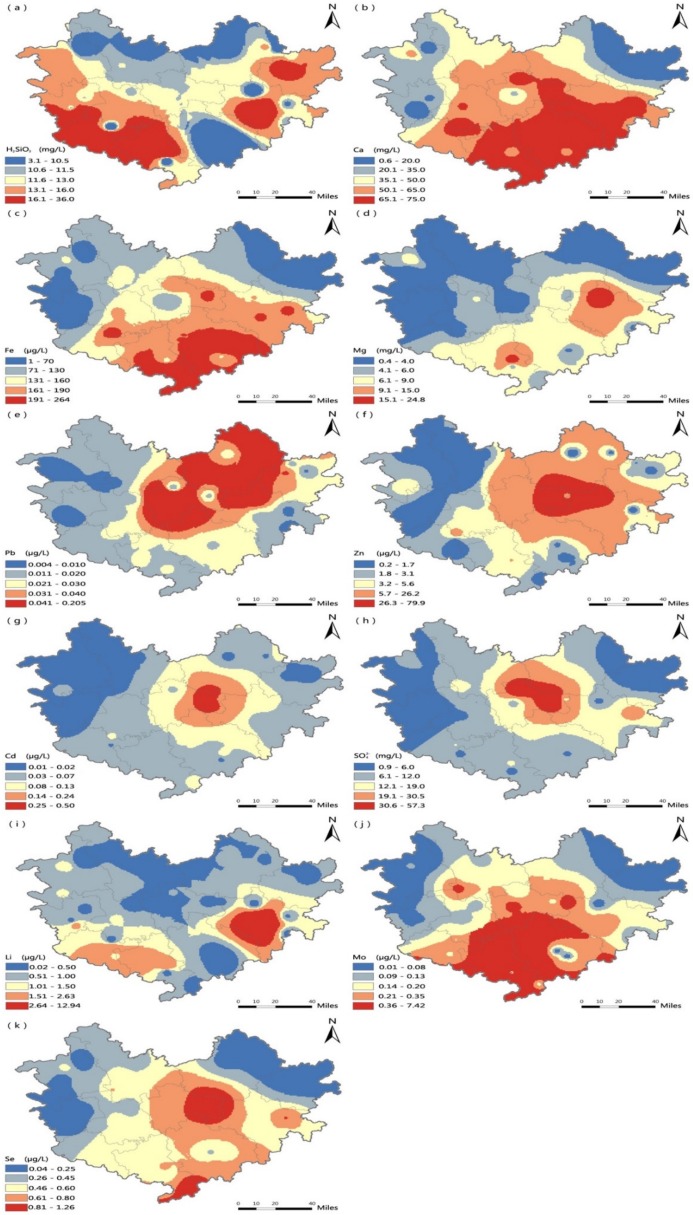
Trace element and mineral levels in drinking water in Hechi: H_2_SiO_3_ (**a**), Ca (**b**), Fe (**c**), Mg (**d**), Pb (**e**), Zn (**f**), Cd (**g**), SO_4_^2−^ (**h**), Li (**i**), Mo (**j**), Se (**k**).

**Figure 5:**
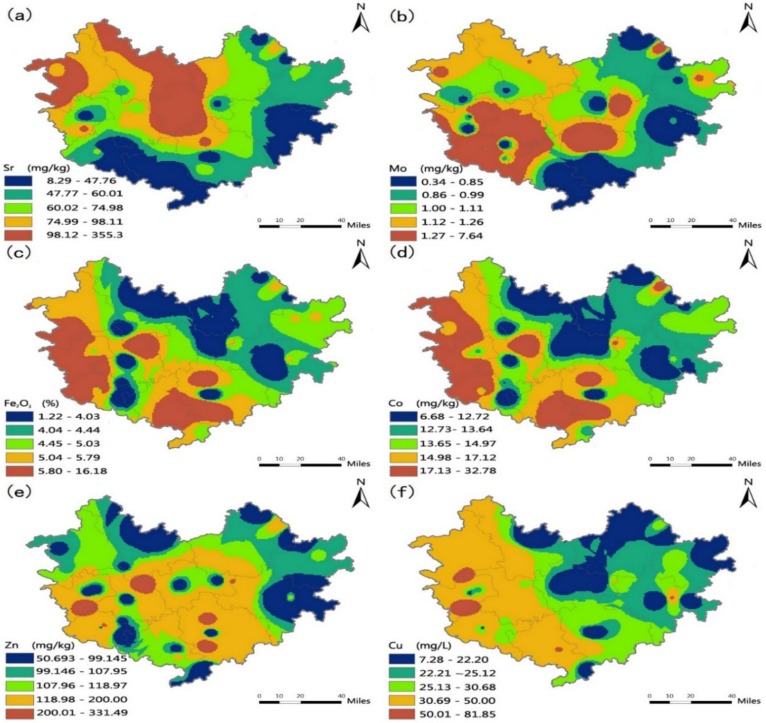
Trace element and mineral levels in soil in Hechi: Sr (**a**), Mo (**b**), Fe_2_O_3_ (**c**), Co (**d**), Zn (**e**) and Cu (**f**).

The spatial distribution depicted in Figure 4 indicates that high standards of H_2_SiO_3_, Ca, Fe and Mg levels in drinking water were mainly distributed in the southern and southwestern areas of Hechi, a distribution that is similar to the higher values of the both longevity indicators shown in Figure 3. In contrast, the higher Pb, Zn, Cd and SO_4_^2−^ level values were mainly distributed in the center and northeastern area of Hechi, opposite to the trends in the centenarian prevalence and longevity index. In addition, Figure 5 shows the distribution of soil trace element levels. The soil values of Sr were higher mainly in the middle and northwestern areas of Hechi and lower in the southwestern area, which was opposite the pattern observed for the two longevity indicators. Higher values of Mo, Fe_,_ Co, were mainly distributed in the southwestern areas, similar to the distributions of the centenarian prevalence and longevity index in Hechi.

## 4. Discussion 

### 4.1. Temporal and Spatial of the Longevity Population in Hechi

Data from the National Population Census in 1982, 1990, 2000, and 2010 showed that Hechi tended to have the leading centenarian prevalence in China and there were stable areas of centenarian clustering in Hechi across the decades. Larger proportions of centenarians tended to be located in areas of longevity in southwestern Hechi, including Bama, Donglan and Fengshan counties, especially Bama, than in other areas. However, the socio-economic index (mainly referring to the average GDP per capital) in the Hechi area was comparatively lower than that of other cities in Guangxi [35,36]. In addition, the socio-economic index in the longevity counties in the southwestern area of Hechi was lower than that of other non-longevity counties from 1982 to 2010. The results indicate that from a long-term perspective, the number of centenarians in Hechi may be more influenced by local environmental factors than by economic factors. From 1990 to 2010, Moran’s *I* values for the centenarian prevalence and longevity index in Hechi were greater than 0.3, with significant spatial autocorrelations. Hechi is located in a relatively remote mountainous area that is isolated from the outside world, suggesting that the natural environment has a significant impact on regional longevity [24,35,36]. Within this context, an excellent natural environment is possibly the key contributor to a large elderly population, especially regarding the distribution of centenarians, as reported by many studies on longevity [3,4]. Apart from the environmental factors, some additional factors might be also important for longevity in isolated mountainous areas. For example, Pes et al. considered the lifestyle such as suitable amount of physical activity in mountainous terrain might be a reason that reduce the associated illness and promote longevity in Sardinia, Italy; and that could be important insight to understand the longevity phenomenon in mountainous areas [37].

### 4.2. Trace Element and Mineral Levels in Drinking Water and the Soil Environment 

Based on this study, the high quality of the drinking water and soil environment may be two important factors contributing to high longevity prevalence in Hechi from the environmental perspective. The average contents of test quality indicators satisfied both Chinese drinking water quality guidelines (GB5479-2006) and WHO guidelines. The drinking water in Hechi exhibited a weakly alkaline range, with a pH of approximately 7.25, and it was rich in trace elements and minerals such as H_2_SiO_3_, Ca, Fe, Na, Mg, Li, Mo and Se and low in heavy metals such as Pb and Cd. As a result, the drinking water in Hechi was generally good. The soil environment in Hechi was also generally good; however, Cd, Zn, Cu, Cr, Pb and Ni levels exceeded the soil standard in some samples (GB15618-2008). The reason is that the high-value areas are located in the northeastern region within the non-ferrous metal vein areas of Hechi. In those areas, the background concentrations of heavy metals were higher than in other areas, as non-ferrous metal exploitation has increased the levels of these metals in soil.

### 4.3. Relationship between Regional Longevity and Trace Element and Mineral Levels in Drinking Water and the Soil Environment

According to correlation analysis, the longevity index in Hechi were significantly correlated with H_2_SiO_3_, Ca, and Fe levels in drinking water and positively correlated with Na, Mg, Li, Mo, and Se levels. In contrast, Pb, Cd, SO_4_^2−^, Mn, and Zn had a negative correlation with the two longevity indicators. Regarding soil, there was a significant negative correlation between Sr and each of the longevity indicators. In addition, there were positive correlations between each of Mo, Fe, Co, Zn, and Cu and the longevity index regarding the results of Pearson correlation analysis, the effect of trace elements in drinking water on regional longevity is more direct and obvious than the effect of trace elements in the soil environment. 

From a drinking water perspective, the alkalinity and trace element and mineral levels of drinking water in Hechi was closely related to the regional longevity observed. Many studies have confirmed that slightly alkaline drinking water, close to the level in the human body and the plasma pH, can keep blood vessels elastic and maintain blood pressure, which is beneficial for the cardiovascular system [16,17]. Regarding the trace elements, mineral H_2_SiO_3_ (silicate) can maintain blood vessel health and prevent arteriosclerosis, coronary heart disease and hypertension [38]. Fe is a component of myoglobin and hemoglobin; Fe is also a component of catalase and peroxidase, affects energy metabolism and may have a positive effect on human health. Additionally, moderate levels of Ca and Mg in drinking water can help to avoid geriatric diseases and conditions such as cardiovascular disease [39], hypertension and osteoporosis and thereby promote longevity [17]. For example, the population in Nicoya Peninsula has been regarded as a longevity region in Costa Rica, and its drinking water was rich in Ca and Mg [40]. The high Ca in the drinking might contribute to strong bones and reduce other associated diseases among the elderly population; and this was regarded as one of the most important contributors for the longevity phenomenon [40]. In addition, in Taiwan, Yang et al. have also confirmed that Ca in drinking water could reduce the risk of death from acute myocardial infarction and the risk cardiovascular diseases [41].

Nonetheless, the relationship between longevity indicators and Se in drinking water was weak compared to that of the other indicators, as shown in Table 5. Previous studies have confirmed that trace elements such as Se can play decisive roles in maintaining human health and preventing many age-related diseases [42,43]. For example, the trace element Se functions as a potent anti-oxidant that protects the body from oxygen free radicals and slows the aging process in humans [17]. In addition, Se and its associated compounds could also help to reduce the incidence of Keshan disease. Thus, appropriate amounts of Se in food and in drinking water may help to promote human lifespan and prevent many diseases. Our results suggest Se in drinking water might not contribute as much as other indicators such as H_2_SiO_3_, Ca, Fe, and Na, in Hechi under the karst landscape. 

In addition, Li is known to exert life-prolonging effects to human [44,45]. In the study, lithium in drinking water in Bama was relatively higher compared with other areas in Hechi and this might be one of the reasons that contribute to its excellent longevity. Other studies have confirmed that Li in drinking water may have positive impacts on regional longevity and human health, consistent with the conclusions of this study. For example, Zarse et al. found that the suitable amount of lithium concentration in the food and drinking water has played the function of anti-aging; reducing mortality and promoting longevity among the selected Japanese municipalities [44]. In addition, Fajardo et al. concluded that the Li in the tap water could play a role of life prolonging effect of human based on the study in Texas [45].

In contrast, heavy metals, such Pb and Cd, in drinking water are known to be toxic pollutants that can negatively impact human health. The present study confirmed the two longevity indicators to be negatively associated with Pb and Cd in drinking water. Pb accumulation in the human body can affect the cognitive functions and contribute to symptoms such as memory loss, insomnia, and blurred vision [46,47], and indirectly reduce the number of centenarians. In addition, Cd can contribute to heart failure, cerebrovascular infarction, osteoporosis and other conditions. Studies have confirmed that heavy metals in drinking water may have negative impacts on regional longevity, consistent with the conclusions of this study. For example, based on the studies of 17 cities, Hao et al. confirmed that Pb may have had negative impacts on longevity in Hainan Province in 2016 [14]. 

### 4.4. Potential Factors That Impact Trace Element and Mineral Levels in Drinking Water in Hechi

The karst landscape plays a significant role in the formation of drinking water in Hechi, contributing beneficial trace elements, in Hechi. The counties with the rockiest areas (greater than 90%) are Fengshan, Bama, and Donglan, with relatively high longevity indicators in the mentioned researches. Many researches have been conducted on the chemical characteristics of the karst landscape. Studies have concluded that rock weathering in the basin areas is the most important contributor to the local water chemistry and carbonate weathering and that the river systems are rich in H_2_SiO_3_, Ca, Na, Fe, Mg, HCO^−3^ and other beneficial trace elements. The rivers in the karst landscape are slightly alkaline. In addition, the landforms of the karst landscape are prone to developing a series of underground caverns due to the strong weathering. For example, the drinking water in Bama is mostly derived from the depths of local caves, absorbing beneficial trace elements in large quantities during the formation processes [48].

As many centenarians live in rocky areas [49], most of the members of the longevity population in Hechi use the underground water source as their primary water supply. These areas do not have much surface water but do have abundant quantities of underground water from underground caverns. For example, in 1982, 1990, and 2000, approximately 75% of the centenarians in Bama County lived in rocky and semi-rocky areas and used underground water as their main water source, and approximately 30% of the underground water areas have sustained approximately 70% of the centenarians in Bama over decades [48]. It can be inferred that the distinctive tectonic settings of Hechi might have long-term impacts on longevity in this region.

## 5. Conclusions

The southwestern areas of Hechi have stably been longevity areas for decades; the core areas of longevity are Bama, Fengshan and Donglan. Based on spatial autocorrelation analysis, the longevity phenomenon in Hechi might be related to the geography of the local natural environment. In this study, drinking water played a more important role contributing to longevity than the soil environment. The drinking water quality was generally good; the drinking water was slightly alkaline; rich in trace elements such as H_2_SiO_3_, Ca, Na, Fe, Mg; and lacking in heavy metals such as Pb and Cd. Thus, the drinking water quality might be the most important contributor to the longevity phenomenon in Hechi. The findings may be useful for developing a healthy aging society based on the environmental aspect, with interactive impacts from both drinking water and soil. Three limitations of this study should be acknowledged. First, this study focused only on environmental factors (drinking water and soil) in one year in Hechi without examining the changes in these factors over a longer timeframe. Second, the quality of the statistical data may have slightly impacted the precision of the results. Third, this study has only collected 40 drinking water samples and 33 soil samples, which may slightly impact on the accuracy of the spatial distribution generated.

## Figures and Tables

**Table 1:** Spatial autocorrelation of two longevity indicators in Hechi.

Index	Year	Moran Index	*Z* Value	*p*-value
Centenarian Prevalence	1990	0.336	1.746	0.080
	2000	0.371	1.949	0.051
	2010	0.597	2.581	0.009
Longevity Index	1990	0.358	1.794	0.072
	2000	0.470	2.127	0.033
	2010	0.522	2.258	0.023

**Table 2:** Statistical analysis of trace element and mineral levels in drinking water in Hechi.

Element	Sample Number	Mean	Median	Minimum	Maximum	SD	National Standard 2006	WHO-Guideline 2004	SRRWQ	
pH	40	7.25	7.29	5.65	8.03	0.38	6.5–8.5	6.5–9.5	94.9	
As (µg/L)	40	0.48	0.12	0.01	13.48	2.14	≤10	≤10	97.4	
Ca (mg/L)	40	47.99	57.18	0.61	91.04	28.73	-	-	-	
Cd (µg/L)	40	0.05	0.02	0.003	0.5	0.08	≤5	≤5	100	
Cr (µg/L)	40	3.23	2.89	0.69	10.2	1.93	≤50	≤50	100	
Cu (µg/L)	40	0.15	0.1	0.01	1.16	0.19	≤1000	≤1000	100	
Fe (mg/L)	40	127.3	145.55	1.81	263.58	77.28	≤300	≤300	100	
K (µg/L)	40	0.66	0.58	0.15	1.74	0.33	-	-	-	
Li (µg/L)	40	1.06	0.4	0.03	12.97	2.11	-	-	-	
Mg (mg/L)	40	5.31	4.18	0.49	24.86	5.12	-	-	-	
Mn (µg/L)	40	1.67	0.09	0.003	44.33	7.4	≤100	≤500	100	
Mo (µg/L)	40	0.33	0.1	0.01	7.45	1.18	≤70	≤70	100	
Na (mg/L)	40	1.93	1.48	0.27	11.53	2.05	≤200	≤200	100	
Ni (µg/L)	40	1.35	1.23	0.11	5.91	1.06	≤20	≤70	100	
P (mg/L)	40	0.15	0.15	0.05	0.26	0.05	-	-	-	
Pb (µg/L)	40	0.03	0.01	0.004	0.21	0.04	≤10	≤10	100	
Se (µg/L)	40	0.45	0.48	0.04	1.26	0.3	≤10	≤10	100	
Sr (µg/L)	40	0.14	0.1	0.01	1.1	0.18	-	-	-	
Zn (µg/L)	40	6.57	1.85	0.23	80.17	14.82	≤1000	≤1000	100	
SO_4_^2−^ (mg/L)	40	9.7	6.44	0.94	57.44	11.48	-	-	-	
H_2_SiO_3_ (mg/L)	40	14.07	11.56	3.09	36.05	8.27	-	-	-	

Note: SD is the standard deviation; SRRWQ is the standard-reaching rate of water quality indicators in Hechi.

**Table 3:** Statistical analysis of trace element and mineral levels in soil in Hechi.

Element	Sample Number	Mean	Median	Minimum	Maximum	SD	GC	SRRHM
Al_2_O_3_ (%)	33	7.58	6.11	1.23	24.59	4.87	-	-
CaO (%)	33	1.09	0.55	0.05	13.28	2.32	-	-
Fe_2_O_3_ (%)	33	5.16	4.85	1.21	16.21	3.01	-	-
K_2_O (%)	33	1.42	1.18	0.15	3.32	0.89	-	-
MgO (%)	33	0.78	0.75	0.08	2.33	0.47	-	-
Na_2_O (%)	33	0.28	0.22	0.09	0.77	0.16	-	-
As (mg/kg)	33	16.53	12.51	4.57	38.78	9.56	40	100
Cd (mg/kg)	33	0.38	0.24	0.05	2.64	0.51	0.3	70
Co (mg/kg)	33	15.3	14.16	6.64	32.83	6.29	40	100
Cr (mg/kg)	33	91.23	73.29	27.76	263.42	58.37	150	91
Cu (mg/kg)	33	30.73	28.62	7.24	82	17.7	50	88
Li (mg/kg)	33	40.6	31.81	9.25	115.52	23.91	-	-
Mn (mg/kg)	33	745.17	317.97	74.9	2617.4	826.91	-	-
Mo (mg/kg)	33	1.18	0.82	0.34	7.79	1.31	-	-
Ni (mg/kg)	33	39.44	28.99	7.09	134.6	30.57	80	94
P (mg/kg)	33	914.73	672.36	411.72	1959.6	485.52	-	-
Pb (mg/kg)	33	33.2	29.99	7.42	88.03	16.82	80	97
S (mg/kg)	33	210.77	208.49	110.62	340.69	53.24	-	-
Se (mg/kg)	33	0.6	0.54	0.3	1.23	0.2	30	100
Sr (mg/kg)	33	69.86	54.5	4.83	355.6	65.12	-	-
Zn (mg/kg)	33	121.46	88.99	50.6	332.15	78.05	200	82

Note: SD is the standard deviation; GC is the grade two criteria of GuoBiao 15618-2008; SRRHM is the standard-reaching rate of heavy metals in cultivated soils of Hechi.

**Table 4:** Trace element and mineral levels in drinking water in longevity and non-longevity counties in Hechi.

Area	pH	As	Ca	Cd	Cr	Cu	Li	Fe	K	Mg	Mn	Mo	Na	Ni	P	Pb	Se	Sr	Zn	SO_4_^2−^	H_2_SiO_3_
B	7.25	0.19	61.33	0.022	2.61	0.08	2.23	157.64	0.77	7.34	0.07	0.20	3.54	1.54	0.19	0.016	0.55	0.20	1.12	9.62	27.41
L	7.31	0.12	48.38	0.03	2.74	0.07	1.17	124.08	0.62	4.42	0.05	0.13	2.37	1.06	0.13	0.01	0.40	0.12	2.03	5.87	18.38
N	7.13	0.12	42.42	0.09	2.24	0.16	0.53	109.62	0.57	2.97	1.38	0.20	0.82	1.35	0.17	0.05	0.51	0.13	11.71	16.48	10.26

Note: B denotes Bama, the county with the highest centenarian prevalence and longevity index; L denotes longevity counties including Bama, Fengshan and Donglan; N denotes non-longevity counties including Huanjiang, Jinchengjiang and Nandan.

**Table 5:** Trace element and mineral levels in the soil environment in longevity and non-longevity counties in Hechi.

Area	Al	Ca	Fe	K	Mg	Na	As	Cd	Co	Cr	Cu	Li	Mn	Mo	Ni	P	Pb	S	Se	Sr	Zn
B	4.41	0.31	4.86	0.68	0.39	0.50	18.32	0.27	15.61	74.52	30.47	35.63	744.59	3.08	37.71	1007.92	25.80	207.09	0.48	33.81	128.11
L	7.72	0.47	6.29	1.20	0.60	0.33	19.84	0.49	17.26	95.44	35.88	46.67	976.88	1.79	48.25	1024.83	35.13	222.79	0.60	52.00	150.44
N	5.73	1.15	3.64	1.52	0.72	0.25	13.45	0.33	12.10	67.21	22.52	35.65	780.23	0.98	29.28	871.08	28.90	205.48	0.52	95.20	103.15

Note: B denotes Bama, the county with the highest centenarian prevalence and longevity index; L denotes Longevity counties including Bama, Fengshan and Donglan; N denotes non-longevity counties including Huanjiang, Jinchengjiang and Nandan.

**Table 6:** Results of the correlation analysis of the centenarian prevalence, longevity index and trace element and mineral levels in drinking water.

Index	Pb	Zn	Cd	SO_4_^2−^	Mn	H_2_SiO_3_	Ca	Fe	Na	Mg	Li	Mo	Se
LI	−0.287	−0.269	−0.239	−0.232	−0.099	0.420 **	0.391 *	0.389 *	0.289	0.229	0.145	0.14	0.134
CP	−0.296	−0.325 *	−0.306	−0.303	−0.196	0.438 **	0.121	0.100	0.205	0.004	0.064	0.007	0.078

Note: * Correlation is significant at the 0.05 level (2-tailed). ** Correlation is significant at the 0.01 level (2-tailed). LI denotes the longevity index in 2010; CP denotes the centenarian prevalence in 2010.

**Table 7:** Results of the correlation analysis of the centenarian prevalence, longevity index and trace elements and mineral levels in soil.

Index	Sr	K	Mo	Fe	Co	Zn	Cu
LI	−0.345 *	−0.275	0.246	0.221	0.219	0.197	0.191
CP	−0.226	−0.187	0.313	0.282	0.282	0.246	0.257

Note: * Correlation is significant at the 0.05 level (2-tailed). LI denotes the longevity index in 2010; CP denotes the centenarian prevalence in 2010.
